# Quantitative imaging of tissue sections using infrared scanning technology

**DOI:** 10.1111/joa.12398

**Published:** 2015-10-29

**Authors:** Samantha L. Eaton, Elizabeth Cumyn, Declan King, Rachel A. Kline, Sarah M. Carpanini, Jorge Del‐Pozo, Rona Barron, Thomas M. Wishart

**Affiliations:** ^1^ Roslin InstituteUniversity of EdinburghEdinburghUK; ^2^ Royal (Dick) School of Veterinary StudiesUniversity of EdinburghEdinburghUK; ^3^ Euan MacDonald Centre for Motor Neurone Disease ResearchEdinburgh, EH16 4SBUK

**Keywords:** image analysis, infrared fluorescent tags, morphometric analysis, quantitative immunohistochemistry, tissue section imaging

## Abstract

Quantification of immunohistochemically (IHC) labelled tissue sections typically yields semi‐quantitative results. Visualising infrared (IR) ‘tags’, with an appropriate scanner, provides an alternative system where the linear nature of the IR fluorophore emittance enables realistic quantitative fluorescence IHC (QFIHC). Importantly, this new technology enables entire tissue sections to be scanned, allowing accurate area and protein abundance measurements to be calculated from rapidly acquired images. Here, some of the potential benefits of using IR‐based tissue imaging are examined, and the following are demonstrated. Firstly, image capture and analysis using IR‐based scanning technology yields comparable area‐based quantification to those obtained from a modern high‐resolution digital slide scanner. Secondly, IR‐based dual target visualisation and expression‐based quantification is rapid and simple. Thirdly, IR‐based relative protein abundance QIHC measurements are an accurate reflection of tissue sample protein abundance, as demonstrated by comparison with quantitative fluorescent Western blotting data. In summary, it is proposed that IR‐based QFIHC provides an alternative method of rapid whole‐tissue section low‐resolution imaging for the production of reliable and accurate quantitative data.

## Introduction

Immunohistochemistry (IHC) is a widely utilised technique in scientific research environments and clinical laboratories (Matos et al. 2010). It is employed to detect the presence and distribution of proteins in their natural state, and in particular disease processes. The concept of immuno‐visualisation has been with us for nearly a century, beginning with immuno‐staining in the early 1930s using a red stain conjugated to benzidin tetraedro (Marrack, 1934), and this was closely followed by the first immuno‐fluorescence (UV‐based) applications in the 1940s (Coons et al. 1941). The key advantage of this technique over other immunological‐based procedures such as Western blotting or flow cytometry is that the anatomical relationship between the protein of interest, its location and spatial distribution are preserved, and therefore expression is not simply calculated as a percentage within a tissue homogenate (Mansfield, 2014).

As a scientific tool, IHC has undergone many subtle alterations in terms of antibody generation and ‘tags’ for visualisation. There are now several methodological variations available that tend to utilise either chromogens such as diaminobenzidine (DAB) or fluorescence‐based tags as labels with visualisation achieved using either light or fluorescence microscopy, respectively. Whilst quantification of standardised IHC experiments should theoretically be as simple as other immunologically based assays such as ELISAs, the reality is that interpretation of results are inherently subjective (Taylor & Levenson, 2006). Many variables can alter the intensity of labelling throughout application of the methodology. However, a major difficulty in obtaining truly quantifiable data is that images are usually collected and scored by human operators, thereby creating potential error and subjective bias. Moreover, inter‐observer variability can add a further barrier to obtaining accurate reliable data for quantification (Rizzardi et al. 2012). In addition, quantification of morphological measurements can provide vital information for researchers, i.e. when investigating the phenotype of gene knockout models or effects of drug therapy on tumour regression (Henriksson et al. 2009; Acehan et al. 2011; Gruber et al. 2013).

Recent technological advances in image capture have allowed digital acquisition, assisting with interpretation and quantification as a function of pixel‐based density or intensity, which in turn corresponds to the abundance of protein detected. However, sophisticated image analysis software such as those with red‐green‐blue (RGB) detectors can be prohibitively expensive. To overcome these issues, free software such as imagej has been developed and can provide semi‐quantitative results (Matkowskyj et al. 2000). For tissue section analysis, this software relies on the operator to import a single representative file comprising of numerous magnified serial images, from a single section, which are aligned and merged using photo‐editing software (such as adobe photoshop). After importation into image analysis software, an area of interest can be selected for semi‐automated analysis of factors such as pixels intensity (Lehr et al. 1999). Although a useful, inexpensive, albeit time‐consuming manner to carry out quantification, data cannot be termed truly quantitative as the signal measurement from conventional IHC labels is not necessarily directly proportional to the intensity of the stain (Watanabe et al. 1996; Matos et al. 2010; van der loos, 2008).

There is therefore a fundamental requirement for a more efficient method of imaging and quantification that would allow an accurate overview of protein expression across large tissue sections (such as whole sagittal or coronal brain sections) without major economic outlay for complex spectral imaging systems (Taylor & Levenson, 2006). An infrared (IR) fluorescent imaging system is a possible alternative to the more traditional image capture and quantification techniques (Kearn, 2004). Whole‐tissue sections can be labelled with IR‐tagged secondary antibodies using a standardised fluorescent IHC protocol, scanned in their entirety with the data acquired quickly, and analysed using the associated propriety software. Quantification of fluorescent IHC (QFIHC), using IR‐tagged secondary antibodies, is expected to be ‘truly quantitative’ as the linear nature of these antibodies has previously been confirmed in the context of quantitative fluorescent Western blotting (QFWB; Eaton et al. 2013) and in cell Western blots (Eaton et al. 2014). Furthermore, multiple separate antigens of interest can be labelled and quantified in the same experimental run when using appropriate imagers. For example, the Odyssey system from LI‐COR can detect two separate wavelengths in the IR spectrum.

In this study, the potential of combining such linear IR‐tagged secondary antibodies with image capture on a LI‐COR Odyssey scanning system for QFIHC was evaluated. It was demonstrated that the quality of ‘low‐power’ images that can be captured using IR‐scanning technology on whole‐tissue sections allows area analyses comparable to that obtained with a Hamamatsu nanozoomer‐XR digital slide scanner. Furthermore, by assessing the distribution of two well‐characterised antigens in murine whole‐brain sections processed with a range of upstream methodologies, it can be confirmed that the sensitivity of the system and the quality of the images obtained are comparable to traditional IHC, and relatively insensitive to processing methodologies. It was confirmed that dual‐labelling of sections can also be carried out and imaged at similar quality and resolution, and that fluorophores in the 700‐nm range are compatible with higher resolution confocal microscopy. Finally, it was demonstrated through comparison with QFWB of microdissected brain regions that QFIHC‐based abundance measurements are likely to be an accurate reflection of tissue protein content.

## Materials and methods

### Animals and tissue

In compliance with the 3Rs, no animals were bred specifically for this project. Where possible all tissue samples used in this current study were derived from existing archived wild‐type (WT) mouse brains or harvested alongside other ongoing experiments. All experimental procedures were approved by The Roslin Institute's Ethical Review Committee, and conducted according to the strict regulations of the UK Home Office Animals (Scientific Procedures) Act 1986 where appropriate.

### Haemotoxylin and eosin (H&E) production and morphological measurements

Wild‐type C57BL/6 mice were culled by decapitation and fixed in 4% paraformaldehyde, cryoprotected in a 20% sucrose solution and snap‐frozen in isopentane prior to storage at −80 °C. Sections were cut at 10 μm and stained with H&E using an automatic stainer (Autostainer XL; Leica) and coverslipper (CTM6 Coverslipper; Thermo Scientific, UK). Images were acquired on the LI‐COR Odyssey imager at 700 nm wavelength and by using Hamamatsu Nanozoomer‐XR digital slide scanner. Areas of anatomical interest were identified and measured by drawing around each region using either imagej software on the sections scanned with the Hamamatsu Nanozoomer or image studio lite (version 5) software with sections scanned using the LI‐COR Odyssey IR imager.

### IHC

To investigate the optimal method of tissue preservation for high‐quality IHC labelling, the following preservation methods were carried out: (i) paraffin‐embedded and paraformaldehyde‐fixed; (ii) frozen and paraformaldehyde‐fixed; and (iii) paraformaldehyde‐fixed followed by cryoprotection. A negative control was included in each experiment by omission of the primary antibody.

- Paraffin‐embedded tissue. Adult CD1 WT mice brain paraffin‐embedded tissue blocks were utilised, sections (6 μm) were selected at the level of the forebrain and hippocampus, and were labelled using a well‐established published protocol (Piccardo et al. 2007). Briefly, slides were de‐paraffinised by immersion in xylene twice and then rehydrated in decreasing percentages of alcohol (99%, 95% and 70% industrial methylated spirit and then in distilled water). Antigen retrieval was carried out by autoclaving the sections in distilled water for 15 min at 121 °C, followed by immersion in formic acid for 5 min and then running water for 15 min. Sections were treated with 70% IMS for 1 min and methanol plus 3% H_2_O_2_ for 10 min to quench endogenous peroxidase activity, followed by immersion in still water for 10 min. Sections were immunostained with either anti‐D2 receptor protein (1 : 500, raised in rabbit) or anti‐Histone H2A.X (1 : 500; Abcam, UK) overnight at room temperature. Sections were washed then incubated for 1 h with a biotinylated secondary antibody (mouse anti‐rabbit 1 : 1000 for the D2‐labelled and H2A.X‐labelled sections). Sections were incubated for 30 min at room temperature with streptavidin solution [Vectastain Elite ABC kit; Vector Labs, Burlingame CA, USA; 10% avidin plus 10% biotin in phosphate‐buffered saline (PBS) + 5% bovine serum albumin (BSA)] and then incubated in a 2% DAB solution plus 0.1% H_2_O_2_ for 2–3 min, or until a colour change was observed. The tissues were counterstained with H&E, dehydrated and coverslipped.
- Frozen tissue – paraformaldehyde‐treated. Adult CD1 WT mice brains sections (15 μm) were fixed in 4% paraformaldehyde and then blocked in 4% BSA + 0.1% Triton‐X/PBS for 1 h at room temperature. The slides were immunolabelled with either anti‐D2 or anti‐H2A.X (same concentration as above and diluted in blocking solution). Post‐incubation, the secondary antibodies were applied (as above) followed by incubation with ABC solution and then application of DAB solution (as above). The tissues were counterstained with H&E, dehydrated and coverslipped.
- Paraformaldehyde‐fixed followed by cryoprotection and storage at −20 °C. Adult C57BL/6 mice brain tissue was fixed with paraformaldehyde and cryoprotected in a 20% sucrose solution overnight before storage at −20 °C. Sections (20 μm) were transferred to a 24‐well plate containing 1 × PBS to carry out the IHC procedure. Endogenous peroxidase activity was quenched in 3% H_2_O_2_ in distilled water, then the tissues were blocked in 5% normal donkey serum diluted in 0.3% Triton‐X/PBS for 1 h at room temperature. The tissues were immunolabelled with either anti‐D2 or anti‐H2A.X (both at 1 : 500 diluted in 0.3% Triton‐X/PBS) overnight at 4 °C. The secondary antibody was applied (same concentration as used in the paraffin‐embedded processing in 0.3% Triton‐X/PBS) for 1 h at room temperature. ABC substrate was applied followed by DAB stain. Sections were mounted on gelatin‐coated slides.

### Immunofluorescence for LI‐COR imaging

The protocols used were identical to those described above for each tissue type, up to and including the primary antibody incubation stage. Two secondary antibodies were applied either the IRDye^™^ 800CW conjugated donkey‐anti‐rabbit IgG (H+L, 1 : 1000) or IRDye^™^ 680RD conjugated goat‐anti‐rabbit IgG (H+L, 1 : 1000), with the former used for labelling of the H2A.X protein and both IR dyes used for labelling the D2 receptor. Sections were incubated for 1 h in the dark, and thoroughly washed in PBS–BSA buffer prior to being coverslipped using Vectashield fluorescence mounting medium (Vector Laboratories, Peterborough, UK).

### Dual‐labelled immunofluorescence

Wild‐type C57BL/6 mouse brains were fixed in 10% formal saline and embedded in paraffin. Sections were cut at 6 μm and de‐paraffinised prior to quenching of endogenous peroxidase activity using 1% H_2_O_2_ in methanol. Sections were washed in PBS containing 0.2% BSA before blocking in 5% normal goat serum. They were immunostained with D2 receptor antibody (1 : 500) followed by incubation with goat anti‐rabbit IR 800CW (1 : 1000; LI‐COR, UK) or goat‐anti‐rabbit IgG IR 680RD in the dark. All subsequent steps were performed in the dark. Nuclei were stained with To‐Pro^®^3 (1 : 500; Life Technologies, UK) or DAPI (1 : 500; Life Technologies) nuclear stain prior for use with the LI‐COR imaging or confocal microscopy, respectively. Slides were mounted using Vectashield mounting medium (Vector Laboratories).

### Image capture from DAB‐stained sections with light microscopy

Labelled sections were analysed on a light microscope (Nikon Eclipse E800; Nikon, Tokyo, Japan). To create the composite section image, overlapping serial photographs of one brain hemisphere were captured at 20 × magnification (approximately 80 pictures total per tissue section). The images were then imported into the Bridge module of adobe photoshop, aligned and merged to produce one composite image.

### LI‐COR imaging

Slides were placed face‐down on the scanner surface, scanned at a wavelength of 700 or 800 nm, or both channels simultaneously with the laser intensity set at 2.0. An initial low‐resolution, low‐quality scan [resolution: 337 μm; quality setting (Q): ‘lowest’] was acquired of the whole slide to identify sections of interest. A smaller box was drawn around the tissue sections, the resolution was set at 21 μm and Q was set at ‘highest’. At these settings, a 1‐cm^3^ area scans in 3 min and 54 s. A single whole‐coronal murine adult brain section scan would be complete in approximately 3 min.

### Confocal imaging of D2 receptor

Paraffin‐embedded sections labelled with the LI‐COR IR dye 680 nm secondary antibody were imaged with confocal microscopy (LSM 710; Carl Zeiss). Serial images were acquired focused on the CA3/dentate gyrus (DG) area of the hippocampus at 20 × and 63 × oil immersion. DAPI nuclei staining was visualised using violet laser at 405 nm wavelength and designated a pseudo green colour for greater contrast with the D2 receptor labelling in red.

### LI‐COR analysis

Image capture and quantification was carried out using image studio ^®^ (version 3.1.4) software. Three specific areas were delineated on the paraffin‐embedded section labelled for the D2 receptor: the cortex; the hippocampus; and the striatum with quantifiable data measured in arbitrary fluorescence units. To assess the stability of the fluorophores, the sections were archived at 4 °C in the dark for 1 year. Following this, the same sections were re‐imaged and quantified using the original image capture settings and quantification criteria.

### image j analysis

Densitometry was performed on the D2 (DAB)‐labelled paraffin‐embedded section to compare relative signal level in the areas described above. The composite image was imported into imagej, threshold levels adjusted to pick up the D2 label only, and the three areas were delineated and a density percentage was calculated.

### QFWB

Three WT BALB/c mice brains were microdissected as previously described (Wishart et al. 2010), with the hippocampal, striatum and cortex removed. Protein was extracted and concentrations determined using a BCA Assay (Pierce) according to manufacturer's instructions as previously described (Eaton et al. 2014). Samples were denatured in NuPage LDS Sample Buffer (4 ×; Invitrogen, UK) at 98 °C, and 30 μg of protein was loaded and run on NuPage 4–12% Bis‐Tris gels (Invitrogen). Gels were run in duplicate in the same electrophoretic tank, with one gel stained for total protein (Expedeon) and the other used to transfer the protein to a polyvinylidene difluoride (PVDF) membrane using the I‐Blot2 transfer system (Invitrogen) on programme 3. Membrane was incubated with Odyssey blocking buffer (LI‐COR) prior to incubation with a rabbit polyclonal antibody directed against D2 receptor protein (1 : 100) overnight at 4 °C. Goat anti‐rabbit IgG (H+L) IR 680RD was applied for 90 min at room temperature (1 : 5000; LI‐COR Biosciences). Membranes and gels were imaged and quantified using the LI‐COR Odyssey scanner and imagepro software, respectively, as previously described (Eaton et al. 2013, 2014).

### Statistical analysis

Unless otherwise stated, data were collected into Microsoft Excel spread sheets and analysed using graphpad prism software. Unless otherwise stated, the data presented in bar graphs represent the mean ± SEM. For all analyses, *P* < 0.05 was considered to be significant. Individual statistical tests used are detailed in the Results or figure legends as appropriate.

## Results

### IR‐based tissue section scanning yields images of suitable quality for accurate morphometric measurements

**Figure 1:**
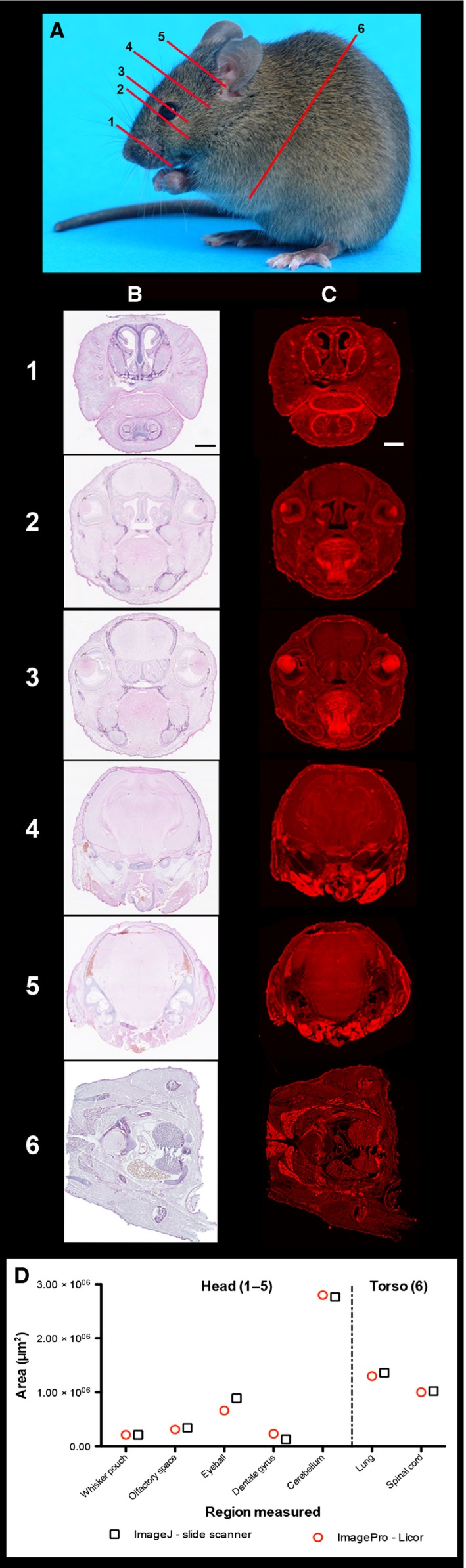
IR‐based tissue section scanning yields images of suitable quality for accurate morphometric measurements. H&E tissue sections can be imaged on an IR scanner. (A) Photographic representation of approximate murine tissue section levels. Representative sections at levels 1–6 are shown in (B) and (C). (B, C) Frozen 20‐μm‐thick tissue H&E‐processed sections were scanned with a Hamamatsu Nanozoomer‐ XR slide scanner (B) and LI‐COR Odyssey imager at 700 nm (C). Areas of anatomical interest within each section, such as: (1) whisker pouches in the nose; (2) emergence of the eyes and olfactory cavity; (3) eye sockets and thalamus; (4) hippocampus; (5) midbrain with cerebellum; and (6) spinal cord. (D) Measurements from each section were obtained using either imagej software applied to Hamamatsu nanozoomer‐XR images or imagestudio lite software on the LI‐COR IR scanner. Scale bars (black for B and white for C): 1 mm.

Conventional morphometric analysis following H&E processing generally requires time‐consuming image tiling microscopy or whole‐tissue section image capture with dedicated slide scanners. Here, it was demonstrated that it is possible to rapidly acquire images from conventional H&E‐stained tissue sections using IR‐scanning technology (Fig. 1). Sections were collected throughout the brain and torso region of a mouse as shown in Fig. 1A. H&E‐processed tissue sections were scanned with a Hamamatsu nanozoomer‐XR digital slide scanner (Fig. 1B) and a LI‐COR Odyssey IR imager (Fig. 1C) to show the image quality that can be obtained. It was also found that by using the LI‐COR‐associated software (image studio lite), it is possible to carry out area‐based measurements to calculate the size of regions/organs of interest. Measurements obtained from the same tissue sections imaged using the two different imaging systems were comparable (Fig. 1D). This suggests that images obtained using IR‐scanning technology are likely to be of suitable quality for conventional morphometric analyses.

### IR fluorophore labelling allows simultaneous low‐resolution dual‐target image acquisition

**Figure 2:**
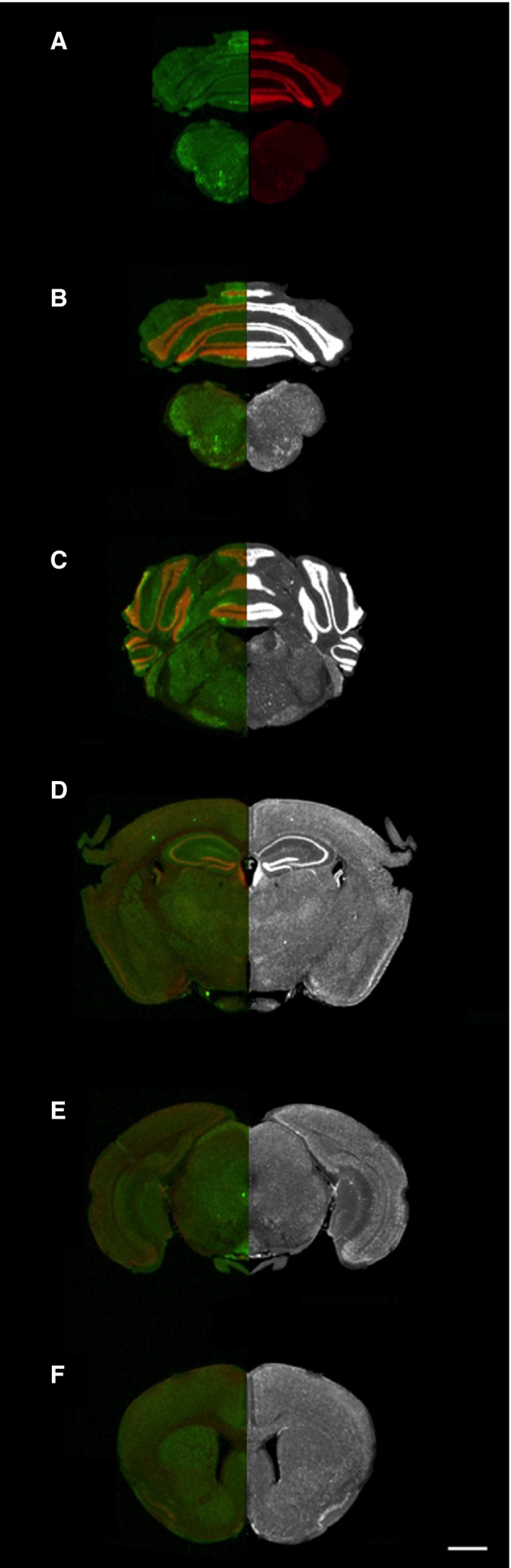
Multiplex images of WT mouse brain sections. Tissue sections probed with the nuclear marker To‐Pro 3 (700 nm channel; red) and the dompaminergic D2 receptor (IRDye 800; green) were captured on the LI‐COR Odyssey IR scanner at maximum quality, 21 μm resolution. (A) Fluorophores compatible with 700 nm (red) and 800 nm (green) wavelengths can be utilised simultaneously. (B–F) Dual‐labelled image is shown on the left‐hand side and a grey‐scale image of the nuclear label is mirrored on the right‐hand panel. Scale bar: 1 mm.

Conventional protein distribution analysis is affected by many of the same problems as H&E‐based morphological examination as mentioned above. Moreover, standard DAB‐based IHC only allows the distribution analysis of a single protein. Therefore, the aim was to determine if multichannel whole‐tissue section protein distribution can be captured with the same quality and speed demonstrated for H&E in Fig. 1. A standard immunofluorescent protocol labelling nuclei and dopaminergic neurons in WT mouse brain sections produced relatively high‐quality images, which were readily reproducible (Fig. 2). Image capture was carried out simultaneously in both channels (700 and 800 nm wavelength), and took approximately 3 min to scan each tissue section in its entirety at the highest quality capture setting. Each label, the nuclear marker To‐Pro3 captured at 680 nm (red channel) and the D2 receptor at 800 nm (green channel), can be viewed independently and quantified separately if required (see Fig. 2A). Furthermore, individual channels can also be viewed in grey‐scale for higher contrast images (Fig. 2B–F). This suggests that IR‐based scanning may provide an alternative methodology for rapid multi‐target image acquisition.

### IR fluorophores are compatible with higher resolution imaging by confocal microscopy

**Figure 3:**
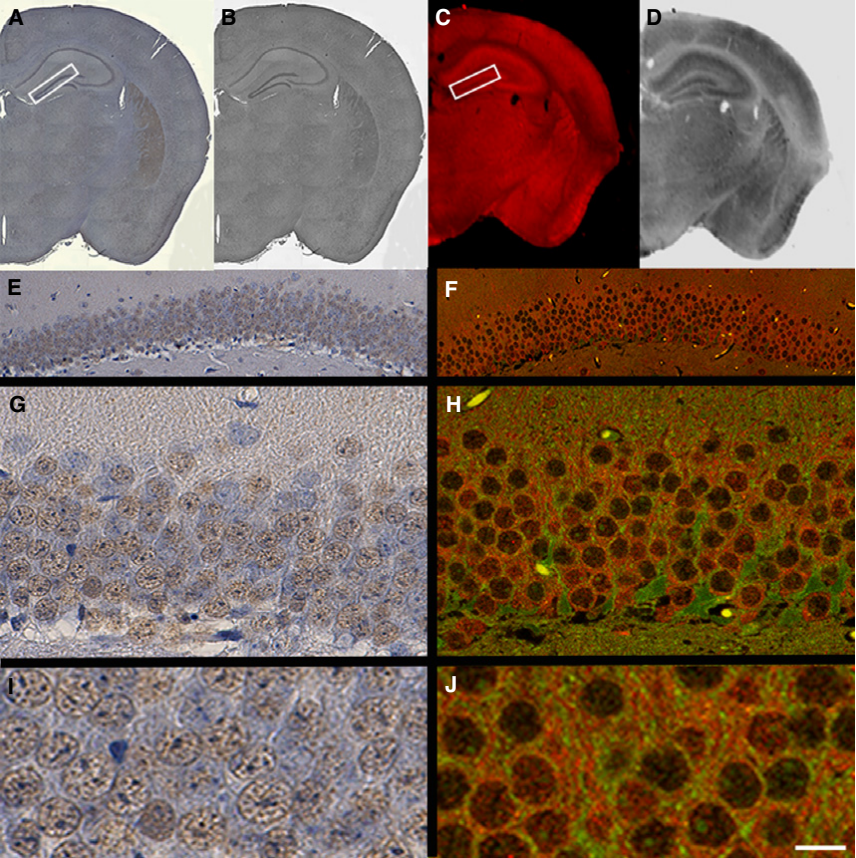
IR fluorophores are compatible with higher resolution imaging by confocal microscopy. Representative tissue sections immunostained for D2 receptor probed with conventional DAB labelling (A, B, E, G, I) or IRDye 680 (C, D, F, H, J). (A, B) Whole coronal reconstructed tissue sections are shown in colour and grey‐scale. (C, D) IR‐captured tissue sections in colour and grey‐scale, respectively. (A, C) An area of the DG outlined with a white rectangular box, subsequently imaged at higher magnification (20 × e and f; 63 × g and h) on either a light or confocal microscope (e and g; f and h, respectively). (I and J) Close crops taken from (G and H) using adobe photoshop. Scale bar: 1 mm (A–D); 80 μm (E and F); 20 μm (G and H); 10 μm (I and J).

**Figure 4:**
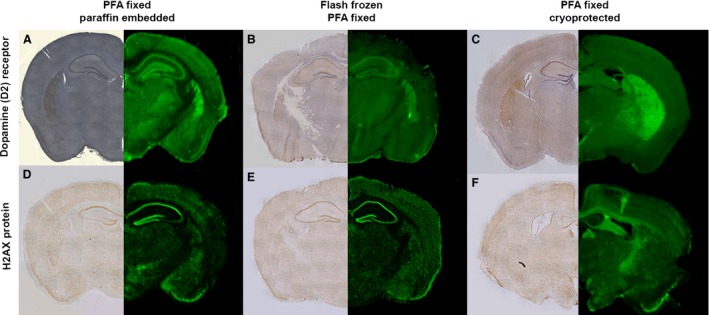
Protein distribution assessed by IR QFIHC is comparable to DAB reporter staining regardless of upstream tissue processing methodologies. The effect of fixation on tissue sections immunostained for two well‐characterised proteins, the dopamine (D2) receptor (A–C) and the nuclear protein H2A.X (D–F). Sections were imaged using either conventional DAB‐based IHC light microscopy or IR‐based scanning. Different tissue fixation methodologies are shown with: (A, D) tissues fixed in paraformaldehyde (PFA) followed by paraffin‐embedding; (B, E) tissues were flash frozen prior to sectioning followed by paraformaldehyde fixation; and (C, F) tissues have been fixed with paraformaldehyde prior to being stored in a cryopreservative and then sectioned. Each panel consists of DAB‐labelled hemispheres (LHS) and IR Dye 800 probed hemispheres (RHS). Scale bar: 1 mm.

Despite the disadvantages and limitations of conventional IHC image acquisition described above, one advantage of DAB‐based IHC is the ability to return to sections and examine areas of interest at higher resolution. Therefore, it was aimed to determine if higher magnification images with greater resolution can be obtained using conventional image capture equipment on tissue sections labelled with IR‐based fluorophores. The Odyssey imager can detect two distinct IR dyes simultaneously: 680 and 800 nm wavelengths. These give indistinguishable patterns of D2 receptor labelling in either wavelength when compared with DAB‐based visualisation on paraffin‐embedded tissue (Figs 3A–D and 4A). Interestingly, as the 680 secondary fluorophore is compatible with the wavelength range of most confocal microscopes, it is possible to obtain higher magnification images of a specific area of interest (i.e. the DG) initially identified on IR‐based QFIHC. Representative images captured at 63 × (oil immersion) on the confocal are compared with ‘high‐resolution’ DAB images captured at 63 × on a light microscope (Fig. 3F,H and E,G, respectively). Importantly, this suggests that not only can whole‐tissue sections be rapidly imaged at low power (3 min acquisition time) to examine overall distribution patterns, but also that higher magnification examinations using other imaging systems, such as confocal microscopy, can also be applied to investigate areas of specific interest.

### Protein distribution assessed by IR QFIHC is comparable to DAB reporter detection regardless of upstream tissue‐processing methodologies

Whilst DAB‐based IHC is part of a standard methodology for assessing protein distribution, many laboratories utilise paraffin‐embedding as part of their upstream tissue‐processing protocols. Paraffin‐embedding typically results in high ‘background’ staining or autofluorescence when combined with conventional fluorescence imaging. Therefore, the aim was to determine if IR‐based fluorophores would be affected in a similar fashion by subtle alterations in upstream tissue‐processing techniques. Here, examples of nuclei identified by H2A.X protein expression and dopaminergic neurones identified by D2 receptor expression were shown, as detected by IHC either conventional DAB staining or IR dye secondary antibody‐based immunofluorescence. This was carried out on tissues preserved by three different methods of pre‐sectioning processing: fresh frozen; paraformaldehyde‐fixed; and cryoprotected or paraffin‐embedded (see Materials and methods).

The pattern of distribution of both target proteins was consistent with previous studies (Kearn, 2004; Ford et al. 2011), and similar across both DAB and IR immunofluorescence detection methods (Fig. 4). D2 receptor protein distribution was widespread throughout the coronal sections, with highest levels of expression visible in the hippocampus, striatum and selected cortical regions with all three‐fixation methods (Fig. 4A–C). Lower levels of expression were also observed in the thalamus, particularly the ventra postero medial and lateral thalamic nucleus as well as the subincertal nucleus, consistent with the published literature (Amenta et al. 1991; Fig. 4A,B). Expression of the nuclear marker H2A.X was widespread throughout the tissue sections (as expected), and visible using both the DAB and fluorescent IHC methodologies (Fig. 4D–F). Similar to the D2 receptor labelling H2A.X protein expression levels appeared more abundant in the hippocampus. However, H2A.X appears more prevalent in the CA1 and the DG with comparatively little labelling observed in the CA2 or CA3 area due to cell density. This therefore suggests that the expression pattern following IR‐based image capture is unaffected by tissue pre‐section processing, which is known to have a dramatic effect on background fluorescence with conventional IHC fluorophores (Buchynska et al. 2008).

### IR‐based image capture is compatible with abundance‐based quantitative regional analysis

**Figure 5:**
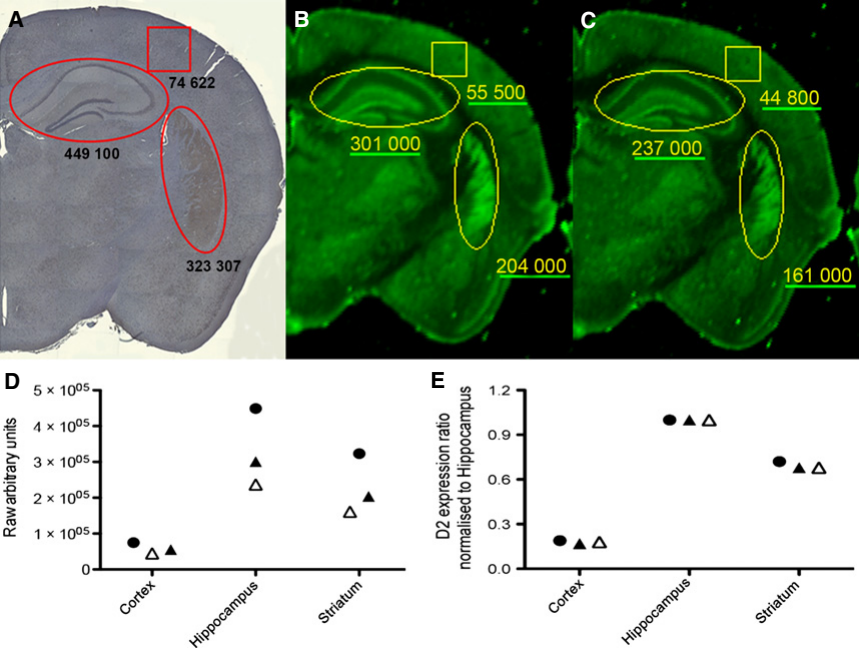
IR‐based image capture is compatible with abundance‐based quantitative regional analysis. Representative images showing D2 receptor labelling in paraformaldehyde‐fixed and paraffin‐embedded tissue sections with (A) DAB, (B) fluorescent IRDye 800, and (C) tissue section from (B), re‐imaged 1 year later. (D) Graphical representation of the raw arbitrary abundance units: a, black circle; b, black triangle; and c, white triangle. (E) Data from (D), normalised to the hippocampus.

Whilst protein distribution may be informative, researchers have continuously sought to be able to analyse protein abundance within distinct brain regions. Here, the quantitative nature and reproducibility of the images obtained was tested by comparing traditional DAB processed sections with IR‐based fluorescence. Quantification of D2 receptor expression was carried out using ImageJ for DAB‐labelled sections, or image studio lite for fluorescently tagged sections captured with the Odyssey imaging system (Fig. 5A–C). The hippocampus, primary somatosensory cortex and striatum were quantified using comparable measuring tools of the same approximate area. Expression was quantified using arbitrary measurement units in both software packages. Similar levels and patterns of expression were observed when comparing the two labelling methodologies, although some variation was seen (Fig. 5D). This may be accounted for with intra‐section normalisation. Here, D2 receptor expression in each brain region was normalised to the area identified with the highest expression values, the hippocampus. Following normalization, it is clear that the relative expression for each brain region is comparable with the two labelling techniques (Fig. 5E).

Interestingly, it was demonstrated that due to the stability of the IR fluorophores it is possible to re‐image and quantify from IR‐labelled sections up to 1 year after initial image capture (Fig. 5C). Although a small decrease in arbitrary fluorescent units was detectable in the sample (Fig. 5D), the decrease in fluorescence intensity was uniform across the brain regions measured, with the expression ratios nearly identical (only 0.01 AU difference; Fig. 5E). This therefore suggests that the IR fluorophores are extremely stable, and that the protein distribution assessment and quantification obtained with IR‐based QFIHC is at least comparable with conventional IHC processes.

### Quantitative analysis by QFWB confirms the accuracy of QFIHC

**Figure 6:**
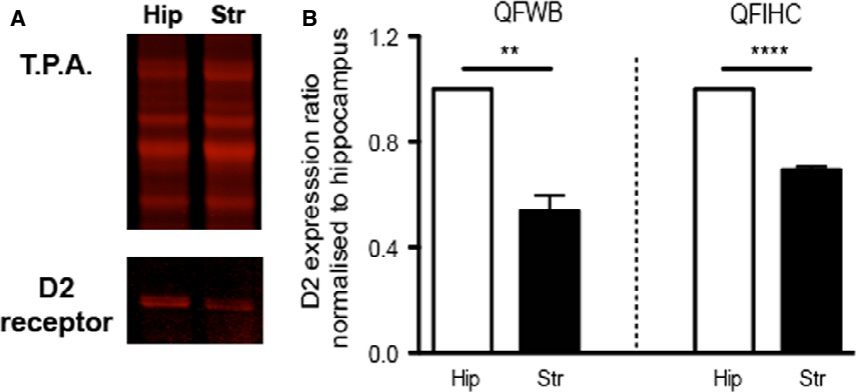
QFWB on microdissected brain regions confirms QFIHC accuracy. (A) Example of QFWB for D2 expression within the microdissected hippocampus (H) and striatum (S) with a total protein loading control (T.P.A.). (B) Bar chart showing ratiometric expression values for D2 receptor in the striatum (black bars) when the hippocampus (white bars) is normalised to 1. Data representative of whole microdissected hippocampal and striatal protein extract (QFWB: LHS) and regional measurements from hippocampal and striatal fluorescence intensity from 20‐μm‐thick tissue sections (QFIHC; RHS – data re‐plotted from Fig 5E). **P = 0.0015, ****P < 0.0001.

The Odyssey IR scanner by LI‐COR was designed to image and quantify Western blots. Whilst IR‐based Western blots are a useful and accurate way of quantifying protein abundance in a complex mixture (Eaton et al. 2013), it does not provide information on distribution. Here, it is attempted to demonstrate the efficacy of IR‐based scanning technology to not only examine protein distribution patterns in a regional manner, but also to quantify the amount of protein present within distinct structures. Whilst it is known that the IR‐based fluorophores are linear in their fluorescence in protein isolates, their behaviour has never been assessed in tissue sections. Therefore, a preliminary investigation was attempted to evaluate whether the values generated by IR‐based IHC are representative of what is present in the tissues examined and therefore determine if the results can be referred to as quantitative. In order to address this, microdissected hippocampal and striatal brain regions were analysed by QFWB (Fig. 6). The upper panel in Fig. 6A shows that the total protein load (30 μg) was uniform for both samples; however, the expression of D2 receptor protein (bottom panel) was greater in the hippocampus than in the striatum as determined by QFWB. Quantitative analysis of the Western blot signal has shown that when the expression ratio of the D2 receptor protein was normalised to the hippocampus, D2 is significantly lower in the striatum (left‐hand panel of Fig. 6B), with the same pattern of expression obtained with QFIHC (right‐hand panel of Fig. 6B). The data are comparable with QFIHC, and it is likely that subtle differences in ratios between QFWB and QFIHC can be attributed to a whole‐brain region homogenate vs. single plane quantification in QFIHC. These data therefore suggest that the values obtained by IR‐based QFIHC are likely to be a realistic reflection of protein abundance within tissue sections.

## Discussion

In recent years, digital capture techniques have advanced image acquisition and analyses to produce truly quantifiable data. In turn, this has allowed scientists and clinicians researching a broad range of conditions to obtain vital data resulting from morphological analyses and measurements of protein abundance (Henriksson et al. 2009; Acehan et al. 2011; Gruber et al. 2013). However, advancements in experimental technologies often require revision and re‐optimisation of routine and reliable methodologies giving rise to reluctance to adapt to such new systems. In this study, it was set out to determine the utility of IR‐based scanning technology in image acquisition for area and protein abundance measurements in whole‐tissue sections.

### Measuring tissue area

Here it was demonstrated that conventional H&E‐processed tissue sections can be imaged with IR‐scanning technologies such as the Odyssey from LI‐COR, and that the associated software is suitable for the measurement of area (Fig. 1). Entire tissue sections can therefore be rapidly imaged at sufficient resolution and quality to carry out area‐based measurements without the need for time‐consuming tiling of images, or expensive dedicated slide scanning technologies. Moreover, the time–cost benefits of whole‐section image acquisition on an IR system (3 min per section) are considerable when compared with traditional serial image reconstruction at 10 × by light microscopy (approximately 120 min). Comparison of measurements from the same tissue sections imaged with a dedicated slide scanner (conventional DAB labelling) and the IR scanner (IR fluorophores labelling) showed little difference between the data recorded (Fig. 1D).

### Measuring protein abundance

In order to determine the quality and resolution of images that can be produced using an IR imaging system, multi‐channel fluorescence IHC was carried out to ascertain the suitability of the images for downstream quantification and publication (Fig. 2). Dual colour images were rapidly and simultaneously acquired on the imager scanning whole coronal murine brain sections within 3 min. This produced images equivalent to approximately 4 × magnification on a conventional microscope. The resolution of these images is suitable for quantification of regional protein abundance or gross morphological measurements. One potential limitation highlighted by others (Hawes et al. 2015) is that sections captured in this manner can only be characterised at the gross regional level. However, it was demonstrated that this potential issue can be overcome by using the IR680 Dye tag, which is captured within the wavelength range of most confocal microscopes as shown in Fig. 3F,H. High‐power confocal images of the 680 IR tag confirm the fluorescent IHC protocol described here yielded labelling of a quality comparable to those tissues labelled using conventional DAB protocol. It was therefore suggested that tissues labelled with an IR680 Dye can be rapidly scanned to provide an overview analysis of regional profiling and expression, but if these images highlight a particular area of interest requiring a higher resolution investigation, the sections will also be compatible with confocal microscopy.

Quantification is becoming a prerequisite of IHC and is more achievable with the advent of digital image capture systems. Densitometry of pixelated areas labelled using conventional streptavidin/biotin complex IHC methodology should only provide a semi‐quantitative analysis as the protein abundance is not necessarily directly proportional to the signal obtained (Taylor & Levenson, 2006). In contrast, labelling with IR Dyes implies that the signal measured should be truly representative of the protein abundance. The fluorescent tag is directly conjugated to the secondary antibody, and it has previously been demonstrated that this produces a truly linear readout when applied to tissue sample homogenates by Western blotting in the authors' hands (Eaton et al. 2013). Analysis of D2 receptor labelling in paraffin‐embedded tissue demonstrated that the pattern of labelling was almost identical between the fluorescent and DAB staining, confirming that the IR secondaries perform as expected in terms of signal localisation (Fig. 5A,B). Furthermore, the ability to quantify from IR dye‐labelled tissue sections yielding quantification in the form of arbitrary fluorescence units from which relative abundance can be convincingly calculated can be demonstrated (Fig. 5). However, as this is a relatively novel application of these IR secondaries, it is possible that there could be factors that impact on their reporting sensitivity, such as tissue penetration, etc. However, by comparing regional expression levels of D2 receptor from whole‐brain tissue sections obtained by QFIHC with the relative expression levels in microdissected brain regions by QFWB, it can be confirmed that the IR fluorophores are indeed likely to be reporting a realistic value for protein expression in QFIHC (Fig. 6).

### Conclusion

This current study reports on a number of interesting observations with regard to the application of IR technology to imaging of tissue sections. Firstly, that fluorescent IHC methodology using IR Dyes provides labelling distribution consistent with traditional streptavidin/biotin labelling with DAB staining. Secondly, that this staining is not grossly affected by the method of tissue pre‐processing. Thirdly, that image capture and subsequent quantification is far less time consuming and costly than other methodologies. Fourth, that H&E sections can be rapidly imaged using the IR scanner and morphometric analyses can be accurately obtained using the associated software. Finally, and perhaps most importantly, that quantification of regional protein abundance by this methodology appears to yield an accurate representation of tissue protein expression values. The Odyssey from LI‐COR has already been put to use in viral detection, cell culture and live animal imaging (Skoch et al. 2005; Leblond et al. 2010; Weldon et al. 2010; Nadanaciva et al. 2011; Kumar et al. 2012), and here it was suggested that it has further merit as a reliable application in whole‐tissue section histological processing and quantification.

## Funding

Work in the authors' laboratories has been supported by grant funding from the Biotechnology and Biological Sciences Research Council (BBSRC) UK Institute Strategic Programme Grant Funding from the BBSRC (RB & TMW) and the MRC (TMW). The funders had no role in study design, data collection and analysis, decision to publish, or preparation of the manuscript.
